# B Cell Tolerance to Deiminated Histones in BALB/c, C57BL/6, and Autoimmune-Prone Mouse Strains

**DOI:** 10.3389/fimmu.2017.00362

**Published:** 2017-03-30

**Authors:** Nishant Dwivedi, Annica Hedberg, Ying Yi Zheng, Indira Neeli, Minoru Satoh, Laurence Morel, Ole Petter Rekvig, Marko Radic

**Affiliations:** ^1^Department of Microbiology, Immunology and Biochemistry, University of Tennessee Health Science Center, Memphis, TN, USA; ^2^Medical Faculty, Department of RNA and Molecular Pathology, Institute of Medical Biology, University of Tromsø, Tromsø, Norway; ^3^Department of Medicine, University of Florida, Gainesville, FL, USA; ^4^Department of Clinical Nursing, University of Occupational and Environmental Health, Fukuoka, Japan

**Keywords:** autoimmunity, antibodies to citrullinated protein antigens, citrullines, B cells, lupus erythematosus, rheumatoid arthritis, autophagy, tolerance

## Abstract

Deimination, a posttranslational modification of arginine to citrulline carried out by peptidylarginine deiminases, may compromise tolerance of self-antigens. Patients with connective tissue autoimmunity, particularly rheumatoid arthritis (RA), systemic lupus erythematosus (SLE), or Felty’s syndrome, present with autoantibodies to deiminated histones (dH), which thus form a category of antibodies to citrullinated protein antigens (ACPA). In general, ACPA are a sensitive diagnostic for RA and may form in response to the release of nuclear chromatin (DNA plus dH) from granulocytes, usually referred to as neutrophil extracellular traps. The aim of this study was to examine spontaneously autoimmune mice for autoantibodies and T cell responses to dH. We compared IgG binding to deiminated and non-deiminated histones (nH) by ELISA and Western blotting in spontaneously autoimmune strains of (NZB × NZW) F_1_ and NZM2410 together with their derivative congenic strains, C57BL/6.*Sle1* and C57BL/6.*Sle1.Sle3*, which display profound autoreactivity against nuclear self-antigens. The splenocyte proliferation against the two antigens was determined in the spontaneously autoimmune (NZB × NZW) F_1_ strain from which other autoimmune strains used in the study were derived. Immunizations with dH and nH were attempted in BALB/c mice to assess their splenocyte response. Splenocytes from BALB/c mice and from autoimmune mice at the time of conversion to autoimmunity proliferated strongly in response to dH, yet serum IgG from autoimmune (NZB × NZW) F_1_, NZM2410, and C57BL/6.*Sle1.Sle3* mice displayed a remarkable bias against binding to dH. At the time of seroconversion, the antibodies already exhibited preference for nH, and only nH were recovered from circulating immune complexes. Analysis of histone deimination showed constitutive deimination in thymic extracts from C57BL/6 and C57BL/6.*Sle1.Sle2.Sle3* triply congenic mice and in spleens of autoimmune triply congenic mice. Our study demonstrates that tolerance mechanisms against dH are intact in BALB/c and C57BL/6 mice and continue to be effective in mice with overt autoimmunity to nH. We conclude that, in contrast to human RA and SLE patients, where we frequently observe autoantibodies against dH, autoimmune mice maintain strong tolerance mechanisms to prevent the development of autoantibodies to dH.

## Introduction

Antibodies to citrullinated protein antigens (ACPA) are diagnostic markers for rheumatoid arthritis (RA) (1) and also arise in other human autoimmune disorders such as systemic lupus erythematosus (SLE) and Felty’s syndrome (2, 3). Citrullines are introduced into proteins by peptidylarginine deiminase (PAD) family of enzymes (4), and much effort has been devoted to learning the circumstances that activate PADs and lead to the PAD-mediated conversion of arginine residues into citrulline residues (5, 6). Several citrullinated antigens have been identified in RA, and a common mechanism has been proposed to account for the generation of citrullinated autoantigens (7–9). The proposed mechanism places particular importance on PAD2 and PAD4, enzymes that are expressed in cells of the innate and adaptive immune system (10, 11). These calcium-dependent enzymes are activated under inflammatory conditions (5). Direct stimuli of PADs include microbial pathogens and pro-inflammatory chemokines and cytokines (5). The enzymes are also activated by sterile inflammatory stimuli, such as cholesterol and urate crystals (8). In fact, it has been argued that any perforation to the plasma membrane could lead to the activation of PADs (12).

One particularly relevant event that is linked to PAD activation and may contribute to the induction of ACPA is a form of granulocyte cell death, which is induced by microbes and inflammatory stimuli and results in the release of nuclear chromatin (5, 13). Such neutrophil extracellular traps (NETs) are considered an innate antimicrobial response because the externalized chromatin is associated with neutrophil granule components such as myeloperoxidase and elastase, which, together with histones themselves, assist in bacterial killing and microbial entrapment (14). In the process of NET release, termed NETosis, PADs gain access to multiple intracellular and extracellular substrates such as histones, filaggrin, fibrinogen, and collagen, which are frequently targeted by ACPA (15). So, it is a prevalent hypothesis that NETosis provides conditions that lead to the production of deiminated (citrullinated) autoantigens that may stimulate cells of the adaptive immune system in the context of an inflammatory response. Moreover, the structural components of NETs, DNA, and histones, also become externalized during NETosis and, in an infection, may become entangled with bacteria and activate the immune system. Interestingly, dendritic cells respond to NETs with the production of interferons and other pro-inflammatory cytokines (16, 17). Other forms of cell death may also have consequences for the induction of autoantibodies, as autoantibodies bind to acetylated histones, a modification of apoptotic chromatin that may elicit autoantibody responses in mice and humans (18, 19).

To provide a mouse model for the study of ACPA, we sought to identify spontaneous mouse models of systemic autoimmunity that would produce autoantibodies to citrullinated histones. Although PAD4 expression parallels the severity of the inflammatory process in mouse models of RA (20), PAD4’s contribution to the production of ACPA has been more difficult to ascertain (21, 22). Because ACPA are difficult to induce in most strains of mice (23), questions have been raised whether mouse ACPA participate in RA pathogenesis at all (22). Human ACPA often react with deiminated histones (dH), and antibody binding to citrullinated histone peptides is a sensitive diagnostic test for RA (2, 24). Because histones are the major substrates of PADs in neutrophils, and dH are built into NETs, we expected that spontaneous anti-histone autoantibodies would preferentially bind to PAD-modified histones. However, we observed that mouse autoantibodies from (NZB × NZW) F_1_ (NZB/W) mice and their recombinant derivative strains, including NZM2410 and C57BL/6J.*Sle1* (B6.*Sle1*) or C57BL/6J.*Sle1.Sle3* (B6.*Sle1.Sle3*) congenics, showed strong preference for non-deiminated histones (nH) over dH by ELISA and Western blot. Thus, even after tolerance to histones was broken and autoantibodies to nH were expressed, autoimmune-prone congenic strains retained B cell tolerance toward dH. B cell binding to dH was repressed, whereas autoantibody binding focused instead on PAD4 substrate arginines. The B cell bias against dH argues that dH remain effective tolerogens in autoimmune mice. In support of this possibility, we observed elevated levels of dH in thymus extracts from B6 and B6.*Sle1.Sle2.Sle3* (B6.TC mice) and spleens of autoimmune B6.TC mice. Our observations suggest that, even in overtly autoimmune lupus mice, central (thymic) tolerance inhibits B cells that react with a deiminated variant of an important nuclear autoantigen. These results point to unexpected intricacies in the murine immune response to deiminated autoantigens. We interpret these results as possible outcomes of PAD expression in antigen-presenting cells.

## Materials and Methods

### Mice

Sera were obtained from B6 mice, as well as from NZB/W, NZM2410, B6.*Sle1*, and B6.*Sle1.Sle3* mice at 6–8 months of age. Tissues were prepared from groups of matched B6 and B6.TC mice of 4–6 months of age. Splenocytes were isolated from 6 BALB/c mice of 4 months of age and 13 NZB/W F1 female mice that were divided into 3 age groups: 6–10 weeks of age, 20–21 weeks of age, and 25–30 weeks of age. The treatment and care of animals were in accordance with the guidelines of the Office of Research, UTHSC, the University of Florida and the Norwegian Ethical and Welfare Board, and the study overall was approved by UTHSC Institutional Animal Care and Use Committee under the protocol #11-164.

### ELISA

**Figure 1:**
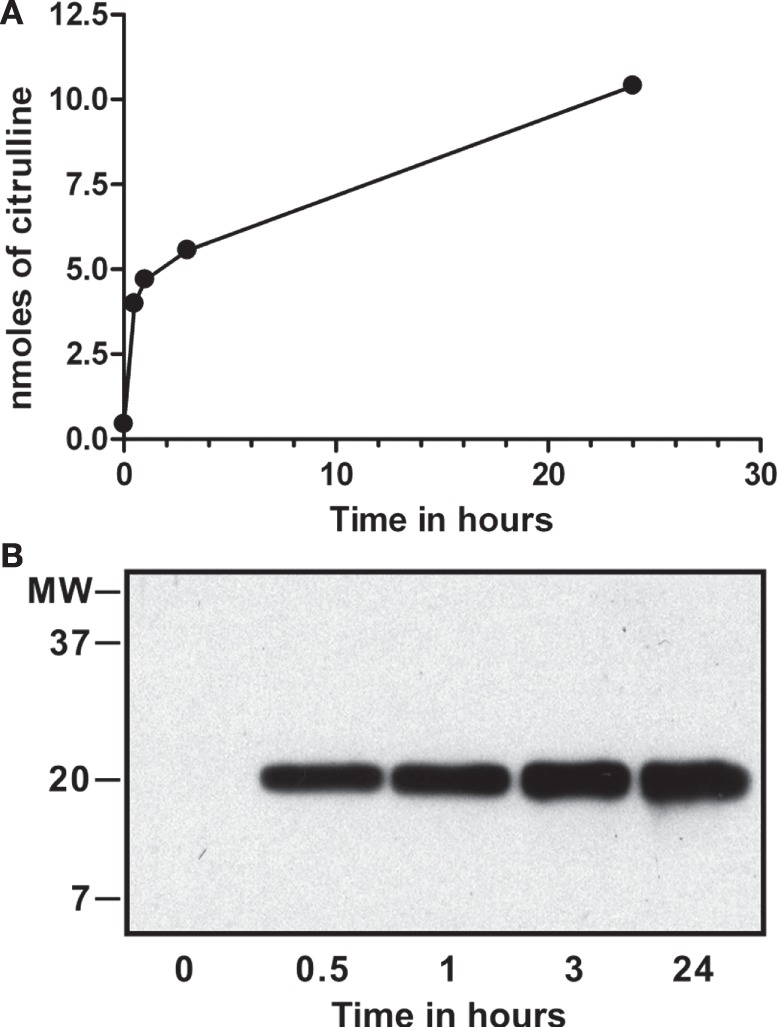
***In vitro* deimination of histones by peptidylarginine deiminase (PAD) 4**. Calf-thymus histones (0.1 mM) were incubated with 0.2 µM of recombinant PAD4 for up to 24 h, and nanomoles of citrulline produced were determined by colorimetry at 595 nm using citrulline standard solution **(A)**. Calf-thymus histones from time points tested above were probed on Western blot with a commercial antibody that reacts against the amino terminus of histone H3 with citrullines at positions 2, 8, and 17 (Abcam, ab#5103). Deimination was detected at each time point except at time = 0 and increased from 0.5 to 24 h **(B)**. The reaction reached a plateau by 24 h, and we calculated that 1.3 citrullines were present, on average, per histone H3 molecule.

For binding assays, we treated purified calf-thymus histones with recombinant PAD4 *in vitro*, as described previously (2, 3). To assess the extent of deimination, we analyzed the progress of the reaction by colorimetry of citrullines and testing the resulting dH by Western blot with an antibody to citrullinated histone H3 (Abcam, ab#5103). The results of this analysis are shown in Figure 1.

Flat bottom, 96 well microtiter plates (Immulon 4HBX; Thermo Electron Corp.) were coated overnight with 5  μg/ml of nH, poly l-lusine, bovine serum albumin (BSA) (Sigma), ovalbumin (OVA) (Sigma), protamine sulfate (Sigma), or dH, as previously described (25). Plates were washed three times with 0.05% Tween-20 in PBS and blocked with 2.5% BSA in 0.02% NaN_3_ and PBS for 2 h. A 1:100 initial dilution of primary sera along with threefold serial dilutions in 1.6% Tween-20 and 1% BSA in PBS were incubated for 1 h in the plates. Then, serum dilutions were removed, and wells were washed with 0.1% Tween-20 in PBS. Alkaline phosphate-conjugated goat anti-mouse kappa (Southern Biotech) was added at 1:1,000 dilution in 1% BSA with 0.05% Tween-20 in PBS for 1 h. Phosphatase substrate (Sigma) was used to develop the ELISA, and OD values were read at 405 nm on a Multiscan Plus plate reader (Labsystems).

Serum antibodies against dsDNA were detected by ELISA exactly as described (26, 27). In short, calf-thymus dsDNA (10 μg/ml in PBS) was coated on microtiter plates (MaxiSorb; Nunc, Copenhagen, Denmark). Sera from mice were diluted twofold from 1:100 to 1:3,200 in PBS containing 0.02% Tween-20 and incubated in wells. ELISA readings were obtained with peroxidase-conjugated rabbit anti-mouse Fc-γ antibodies at 405 nm.

### *Ex Vivo* Tissue Lysate Preparation

Seven-month-old B6.TC autoimmune female mice and age-matched control B6 IgH^a^ were dissected to recover a portion of spleen, bone marrow, kidney, and liver. Thymi from 4- to 6-month-old mice were similarly obtained. Tissue was cut, minced with scissors, and crushed between two sterile frosted glass slides. Dissociated tissues were washed in PBS (without Ca^++^) and centrifuged at 5,000 × *g* for 5 min to pellet cells. Cell pellets were mixed with lysis buffer (65mM Tris pH 7.2, 2%SDS, 10% glycerol), containing protease inhibitors. To test for dH in tissue lysates, equal amounts of total protein were analyzed by Western blotting, as described below.

### Western Blot

For Western blot analysis, proteins were resolved on 15% SDS-PAGE and transferred to nitrocellulose. Membranes were blocked in 5% BSA in 0.1% Tween-20 in TBS (TBST) overnight at 4°C. Subsequently, the membranes were incubated with sera at 1:500 dilution in TBS containing 2.5% BSA, 1% NP-40, and 0.1% SDS. After 2 h of incubation, membranes were washed with 1% NP-40 in TBS. Anti-mouse IgG–HRP was used for detection at 1:20,000 dilution in TBST for 1 h, and blots were developed using chemiluminescence (PerkinElmer).

Peptide inhibition assays included a preceding step, in which 3 µg of 20-mer peptides, both matching the amino terminus of H3 but either containing arginines or citrullines at positions 2, 8, and 17, were incubated with 1:300 dilutions of mouse sera for 1 h prior to use in binding to dH and nH on the membrane. Results of these Western blots were quantitated by infrared emission of secondary anti-mouse IgG antibodies (Odyssey). Separately, autoimmune sera were treated with DNase1 prior to Western blotting. Briefly, 400 μl of a 1:200 dilution of sera were incubated with 20 units of DNase1 (New England Biolabs) for 1 h at room temperature to limit the possibility that DNA–anti-DNA complexes present in sera contribute to the observed histone binding. Following this incubation, the sera were diluted 1:500 in Western blot binding buffer.

To probe for deiminated histone H3 (dH3) in B6.TC and B6 mice, equal amounts of tissue lysates were resolved on 12% SDS-PAGE and transferred to nitrocellulose. Membranes were blocked with 5% BSA in TBST for 30 min and incubated with anti-dH3 anti-citrullinated histone H3 rabbit antibodies (Abcam, ab#5103) overnight at 4°C. Membranes were washed and incubated with HRP-conjugated goat-anti-rabbit secondary IgG antibody for 1 h at room temperature, washed three times in TBST, and twice in TBS alone. The HRP activity was detected as above.

### Splenocyte Proliferation Assay

BALB/c mice were boosted twice with 100 µg of total histones (dH or nH) or 100 µg OVA, 14 and 2 days prior to the splenocyte proliferation assay. Ninety-six well tissue culture plates (Corning Incorporated) were filled with 100 µl aliquots of 100 µg/ml (or threefold serial dilutions) of dH, nH, or OVA in RPMI 1640 (Mediatech Inc.) supplemented with 10% FBS. Splenocytes were isolated and resuspended in RPMI with 10% FBS at 1 × 10^6^ cells/ml. One hundred microliters of cell suspension was added to each well, and plates were incubated at 37°C in 5% CO_2_ for 72 to 96 h. Tritiated thymidine (1 μCi/well) was added for the last 17 h of incubation. Plates were harvested onto glass fiber filters, and thymidine incorporation was assessed by scintillation counting. Splenocyte proliferation assays were also performed using female NZB/W mice purchased from the Jackson Laboratory (Bar Harbor, ME, USA). Splenocytes were collected from NZB/W mice of different ages. Following red blood cell lysis, the splenocytes were resuspended in DMEM-10 media with 10% FCS and 10,000 U/ml penicillin and 10 mg/ml streptomycin. One hundred thousand cells were incubated with dH or nH (20 μg/ml of protein) in triplicate wells. Tritiated thymidine incorporation (1 μCi/well) was measured after 20 h, 3 or 6 days by liquid scintillation, as described (28, 29).

## Results

### Spontaneously Arising Anti-Histone Autoantibodies

**Figure 2:**
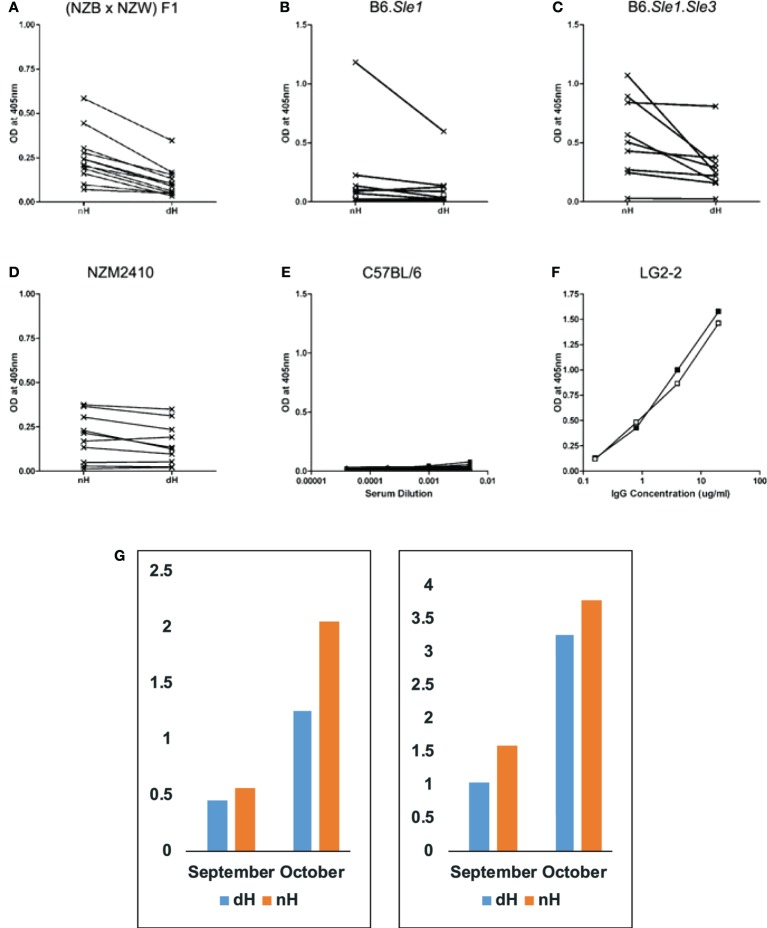
**Binding of IgG from autoimmune-prone and control mice to non-deiminated histones (nH) and deiminated histones (dH)**. Sera from NZB/W **(A)**, B6.*Sle1* **(B)**, B6.*Sle1.Sle3* **(C)**, NZM2410 **(D)**, and control B6 **(E)** mice were tested for IgG binding to nH and dH. Complete binding curves were obtained, and OD values for a single dilution were plotted in panels **(A–D)**. We plotted data from 1:1,000 dilutions in panels **(A–C)** and from the 1:200 dilution in panel **(D)**. Absorbance values for each serum corresponding to IgG binding to dH and nH are shown and are connected by a line indicating the pairs of data for the binding of each serum to the two antigens. Significance of the readings was tested by paired, one-tailed *T*-test. Binding to dH was significantly less than to nH for NZB/W (*p* < 0.0001), NZM2410 (*p* < 0.016), and B6.*Sle1.Sle3* (*p* < 0.016). The binding of B6.*Sle1* IgG tended to be lower to dH (*p* < 0.10). IgG from mice with systemic lupus erythematosus susceptibility genes showed preferential binding to nH. In comparison, control B6 mice showed negligible binding to either form of histones **(E)**. As control for equal coating of Ags, we used LG2-2, a mouse anti-histone H2B mAb **(F)**, whose epitope does not include any residues that are substrates for deimination (31). Thus, the binding curves for dH (filled symbols) and nH (open symbols) are nearly superimposable. Individual mice were followed over time **(G)**, to observe the initial development of anti-histone autoreactivity. Two NZB/W mice that first showed anti-histone reactivity at 4 months of age (September) reacted more strongly to nH than to dH, and the preferential antibody binding was maintained at 5 months of age (October). The sera were diluted 1:300, and the measurements were performed three times with consistent results. The Y-axis displays values of optical density measured by ELISA.

**Figure 3:**
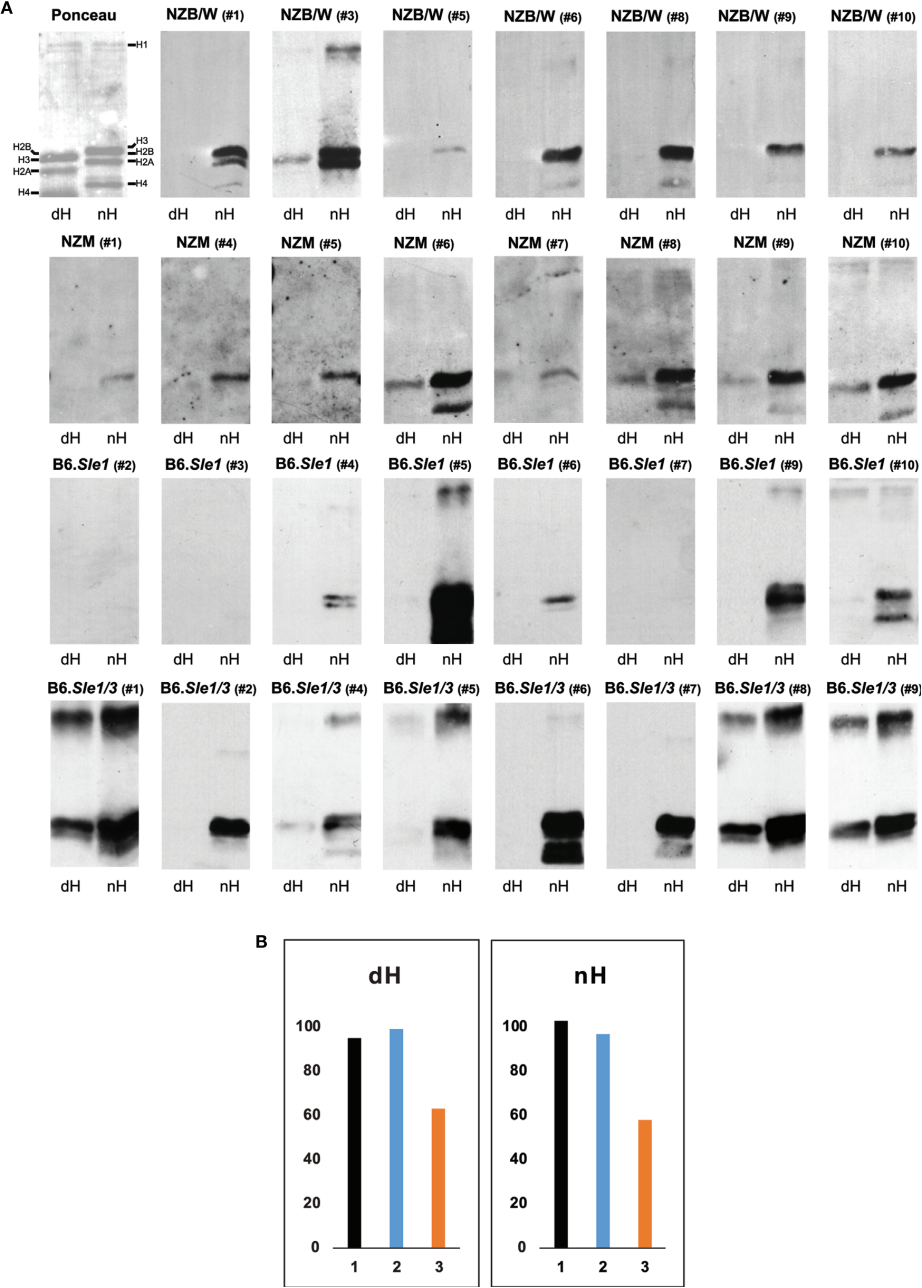
**Western blot of IgG to deiminated histones (dH) and non-deiminated histones (nH)**. Equal amounts of dH and nH were resolved on SDS-PAGE, transferred to nitrocellulose, and stained with Ponceau red (upper left panel). The stained bands migrating with the mobilities of core and linker histones are indicated along the margins. Note that due to the replacement of the positively charged arginine by the neutral citrulline, the electrophoretic mobility of certain core histones is increased in the dH sample, indicating nearly complete modification by peptidylarginine deiminase 4. **(A)** Strips of membrane containing nH or dH were probed with NZB/W, NZM2410, B6.*Sle1*, and B6.*Sle1.Sle3* sera at 1:500 dilution and developed with anti-mouse IgG-horseradish peroxidase. Autoimmune-prone mouse sera bound nH in preference to dH. The experiments were performed at least three times with consistent results. **(B)** To explore whether the binding to histones on the membranes is equally sensitive to inhibition by 20-mer peptides matching the H3 amino terminus and containing arginine residues (nH peptide; orange bars) versus citrulline residues (dH peptide; blue bars) at positions 2, 8, and 17, we preincubated an NZB/W serum that showed binding to both nH and dH (black bars) with either peptide, as described in Section “Materials and Methods,” and carried out the Western blots. The nH peptide was a more effective inhibitor of binding to both histones than the dH peptide. The *Y*-axis indicates binding intensities in units of infrared emission (IE).

To assess the production of anti-nH/dH autoantibodies in mice that spontaneously develop antinuclear autoantibodies, we tested sera from NZB/W and their recombinant inbred derivative strain NZM2410 for binding to nH and dH. In addition, we tested the contribution of lupus-predisposing genetic intervals *Sle1* and *Sle3* that were back-crossed from the NZM2410 onto the B6 background (B6.*Sle1* and B6.*Sle1.Sle3*). *Sle1* is a locus that breaks tolerance to chromatin, whereas *Sle3* affects functions of myeloid cells (30). The parental strains, NZB and NZW, have distinct MHC, H-2^d^ and H-2^z^, respectively. The lupus-predisposing H-2^z^ was maintained in the NZM2410 congenics, whereas the *Sle1* and *Sle3* congenics have the H-2^b^ from B6. Antibody binding to dH and nH was assessed by ELISA (Figure 2) and Western blot (Figure 3). As controls, sera from age- and sex-matched B6 mice were used.

By ELISA, NZB/W (Figure 2A), B6.*Sle1* (Figure 2B), B6.*Sle1.Sle3* (Figure 2C), and NZM2410 (Figure 2D) sera showed preference for nH over dH. This preferential binding was statistically significant for NZB/W, NZM2410, and B6.*Sle1.Sle3*, as assessed by paired, one-tailed *T*-test. Binding differed for different mice and dilutions but, in general, binding to nH was stronger than the binding to dH. We also confirmed the additive effect of *Sle1* and *Sle3* loci, as the B6.*Sle1.Sle3* combination resulted in greater absorbance values relative to the B6.*Sle1* mice. In parallel assays, sera from B6 mice (Figure 2E) showed no reactivity to histones. Both dH and nH were present in equivalent concentrations on the plates, as shown by the nearly identical binding of the LG2.2 monoclonal antibody (Figure 2F) whose epitope, the first 13 amino acid residues of histone H2B, is identical between nH and dH (31).

To examine the possibility that antibodies to dH arise first but are replaced by antibodies to nH, we collected mouse sera over time to identify mice during the conversion to anti-histone autoimmunity. In Figure 2G, we show that binding preference to nH over dH was present at an early time when anti-histone reactivity first appeared. This result indicates the two reactivities arise jointly, rather than in succession, as might be predicted by epitope spreading.

To dissect the preferential binding to nH, we used Western blotting. The stringency of binding was increased by including both SDS (0.05%) and NP-40 (0.5%) in the binding buffer. Indeed, under these conditions, the binding of serum antibodies from NZB/W, NZM2410 B6.*Sle1*, and B6.*Sle1.Sle3* mice to dH was weaker relative to the binding to nH, such that many of the sera bound exclusively to nH (Figure 3A). A variety of binding patterns were observed, including exclusive binding to one or two core histones. Binding to dH3 was observed most often, whereas binding to deiminated H4 or H2A was rare. In addition, binding to a band with the mobility of the deiminated linker histone H1 was observed in several blots. Overall, binding was more biased toward nH over dH, although some IgG dH was also observed in individual NZM2410 and B6.*Sle1.Sle3* mice. In no instance did binding to nH exceed binding to dH.

To examine the possibility that the binding to dH represented a truly separate population of antibodies, we conducted inhibition experiments using 20-mer peptides that matched the amino terminus of histone H3 and either contained arginines or citrullines at positions 2, 8, and 17 (Figure 3B). We observed that the arginine-containing peptide (orange bars) was a more effective inhibitor of binding to both nH and dH, relative to the citrulline-containing peptide (blue bars), suggesting that the antibody binding to either antigen reflects antibody specificity for nH and that the binding to dH likely represents cross-reactivity due to shared epitope structure.

### Splenocyte Proliferation

**Figure 4:**
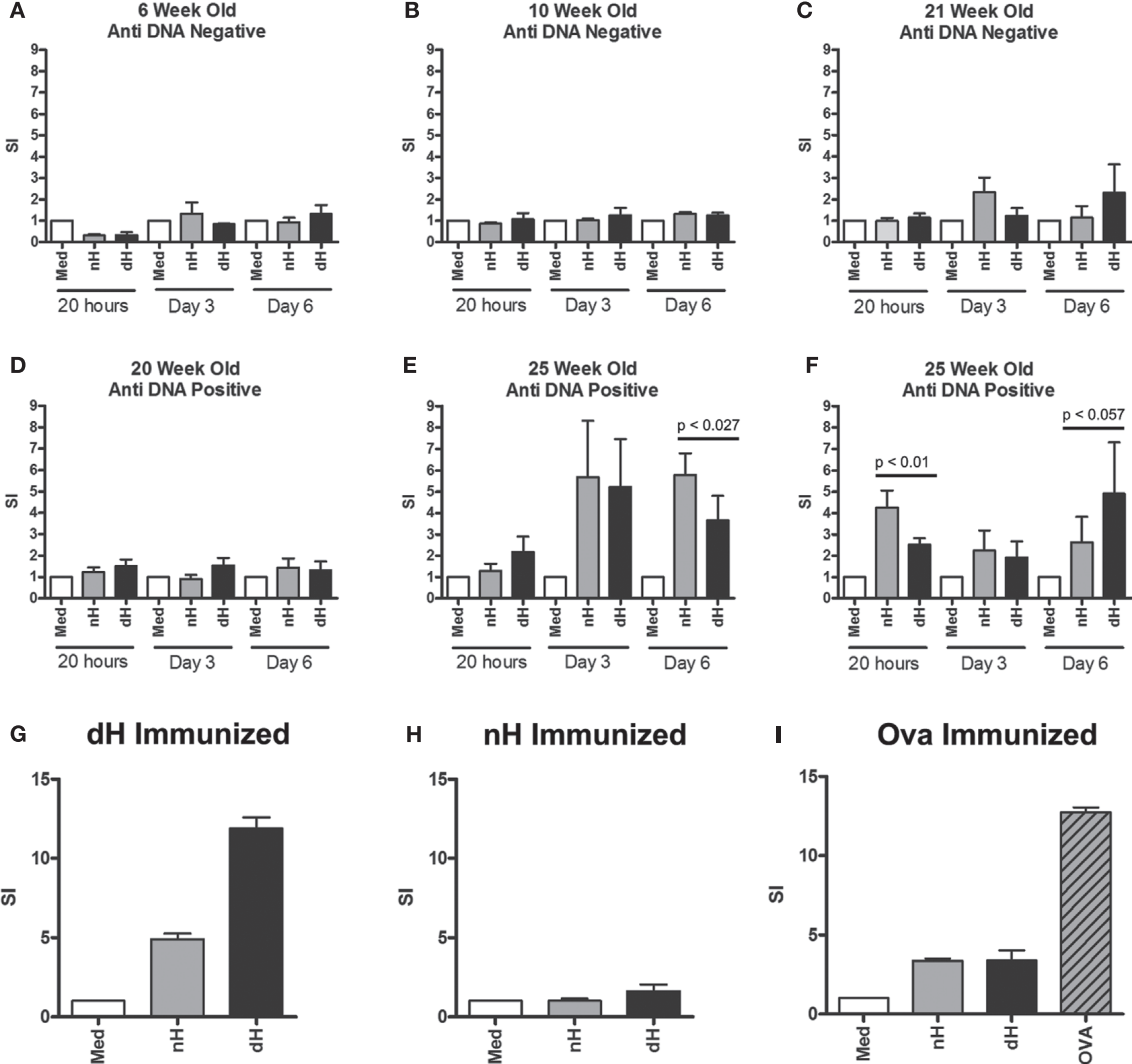
**Proliferation of splenocytes from NZB/W and immunized BALB/c mice**. Splenocytes derived from NZB/W mice of different ages were tested for dsDNA binding by ELISA **(A–F)**. In parallel, splenocytes were stimulated with deiminated histones (dH) or non-deiminated histones (nH) *in vitro*, as indicated. Proliferation was assessed following 4 h, 3 and 6 days in culture, and the response was determined by [^3^H] thymidine incorporation. In addition, splenocytes from mice immunized with dH **(G)** or nH **(H)** were tested for proliferation in response to dH or nH, or ovalbumin **(I)** as control. The results are presented as stimulation indices (SI) that were calculated from mean cpm values of triplicate wells. The significance of differences between samples was determined by unpaired *T*-test, and the *p* values are indicated.

We asked whether T cells from autoimmune mice also recognize dH by using NZB/W mice that spontaneously develop an autoimmune response against nuclear Ags, including DNA and histones (32). Splenocytes from NZB/W mice of different ages were tested for proliferation in the presence of dH or nH. At 6–10 weeks of age, prior to any measurable anti-DNA reactivity, the splenocytes did not proliferate in response to either form of histone (Figures 4A,B). At 20–21 weeks of age, anti-DNA autoantibodies could be detected in the sera of some NZB/W mice (indicated at the top of each panel), and splenocytes from these mice showed low levels of proliferation in response to nH and dH (Figures 4C,D). Thus, splenocyte responses to histones arose in parallel with, or slightly prior to, humoral responses to DNA.

Twenty-five-week-old NZB/W mice with established autoimmunity showed splenocyte proliferation in response to dH and nH (Figures 4E,F), suggesting the presence of histone-reactive T cells in the spleens of autoimmune mice. Although some mice showed a tendency to preferentially respond to dH, others preferred nH, as shown by data from two of the analyzed mice. Notably, preference could switch, depending on the length of stimulation (Figures 4E,F), suggesting the presence of a limited number of T cell clones with distinct specificities and growth characteristics. Because proliferation generally showed a bias for dH or nH rather than being equal, we infer that epitopes containing arginines or citrullines were both presented by the MHC and recognized by T cells in splenocytes.

To examine the ability of dH to drive a T cell response, we examined T cell proliferation *in vitro*. Splenocytes from BALB/c mice immunized with dH proliferated during incubation with dH (Figure 4G) to comparable extent as splenocytes from mice immunized with OVA and incubated with OVA (Figure 4I). By contrast, splenocytes from BALB/c mice immunized with nH showed no enhanced proliferation regardless of whether they were incubated with dH, nH, or media alone (Figure 4H).

### Spleens of Autoimmune Mice Have Increased Levels of dH

**Figure 5:**
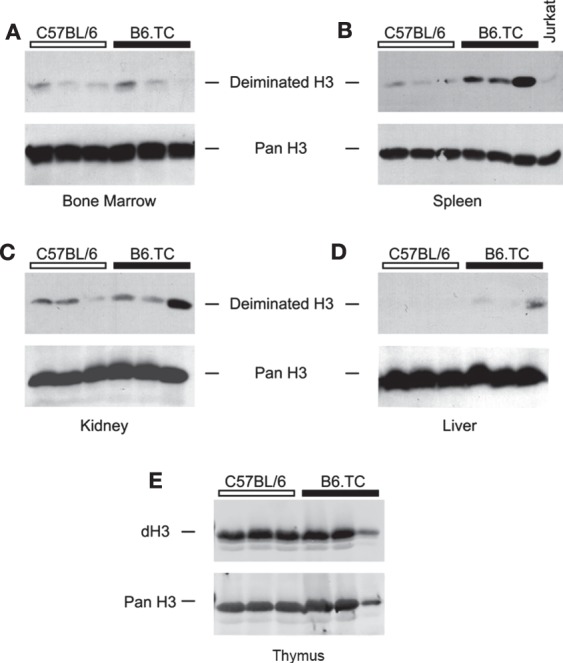
**Western blot detection of deiminated histone H3 (dH3) in mouse tissues**. Equal amounts of freshly prepared lysates from bone marrow **(A)**, spleen **(B)**, kidney **(C)**, liver **(D)**, and thymus **(E)** of autoimmune B6.TC and control B6 mice were blotted onto nitrocellulose membranes and probed with anti-dH3 antibody. Tissues from three mice were tested in each group. As control, Jurkat lymphoma cells were used **(B)**. The same blots were also re-probed with an antibody that recognizes total histone H3 (Pan H3) to confirm equal loading of lysates.

To test for the presence of dH *in vivo*, we prepared tissue lysates of bone marrow, spleen, liver, kidney, and thymus from B6.TC mice and probed them with anti-dH by Western blot. The bone marrow lysates of autoimmune B6.TC mice and control mice at 7 months of age did not appreciably react with antibodies to dH3 (Figure 5A). By contrast, spleens of B6.TC mice had clearly increased levels of dH3 as compared to B6 controls or lysates of the Jurkat lymphoma cells (Figure 5B). These results provide a qualitative estimate rather than a precise measure of deimination. This is by necessity, as the cellular composition and disease process may affect histone deimination in a complex manner in a tissue such as the spleen. The overall amounts of histone H3 were similar in all samples, as indicated by the equivalent immunoreactivity of an anti-H3 antibody. Therefore, the spleens of autoimmune B6.TC mice contained increased quantities of dH3. In addition, the kidneys and the liver from individual autoimmune mice exhibited increased levels of dH3 (Figures 5C,D). Importantly, thymic extracts from 4- to 6-month-old B6 and B6.TC mice clearly showed constitutively elevated levels of dH (Figure 5E).

## Discussion

In this study, we observed that autoimmune mice exhibit a striking B cell bias toward binding nH over dH, a bias that is present at the earliest stages of anti-histone autoimmunity (Figure 2G). In 28 of 28 autoimmune NZB/W, NZM2410, B6.*Sle1*, and B6.*Sle1.Sle3* mice that made IgG anti-histone antibodies, preference was invariably in favor of nH (Figure 3A). Even more strikingly, in 18 animals that produced antibodies to nH, immunoblotting could not detect antibodies to dH. To exclude the possibility that anti-dH antibodies are only transiently expressed, or ensconced in immune complexes, we carried out longitudinal antibody-binding assays that consistently revealed preferential or exclusive binding to nH (Figure 2G). These results indicate that, even in mouse strains that spontaneously convert to autoimmunity, deimination reduces IgG binding to histones, and autoantibody binding is focused on arginine-containing epitopes that are absent from dH. It follows that dH remain effective tolerogens even after development of autoimmunity in the tested mouse strains. We conclude this is an important characteristic of autoimmune mice, and we propose that a more detailed comparison with autoimmune disease patients may shed light on the induction of autoimmunity. Moreover, we argue that the specific regulation of PAD4 underlies these results.

Peptidylarginine deiminase 4 is most abundant in granulocytes and other cells of the innate immune system. However, PAD4 is also expressed in another immunologically relevant context. In elegant studies, Ireland and Unanue described the fact that antigen-presenting cells in mice express PAD4 and PAD2 in a compartment that is regulated by proteins in the autophagy pathway (33). The deiminase activity is constitutively expressed in dendritic cells and macrophages, whereas it is inducible in B cells by stress or stimulation through the Ig antigen receptor (33). The authors reported that arginine residues in antigenic peptides are converted to citrullines, and that T cells respond to target antigenic epitopes containing citrulline. This mechanism was demonstrated by using foreign antigens, in their case, hen egg lysozyme that was administered in a conventional immunization. The resulting T cell clones bound preferentially, or even exclusively citrullinated lysozyme peptides. Our results support this mechanism because splenocytes from BALB/c mice proliferated as vigorously against dH as against OVA (Figure 4G versus Figure 4I). By contrast, splenocytes from NZB/W mice, after these mice had converted to autoimmunity, showed comparable proliferation to either antigen (Figures 4E,F). This fundamental difference in outcome points to differences in immunized versus spontaneous autoimmune responses to nH.

As further shown by Ireland and Unanue, autophagy induction in B cells is necessary for their inducible expression of PAD4 activity associated with antigen processing (33). Conversely, a transient or sustained impairment of autophagy in B cells could provide conditions that would support the presentation of histone epitopes lacking citrullines. Under these conditions, B cells would express peptide epitopes that could act as neo-antigens for T cells and solicit T cell help. Autophagy has been linked by genetics to autoimmunity (27). A contribution of the autophagic processes to autoimmunity is consistent with the deficient or impaired functions of ATG5 (and other components of non-canonical autophagy) in SLE, but the mechanism for this relationship is unclear (27, 28). Our data suggest that effective autophagy may be required to maintain certain aspects of immune tolerance in mice.

However, an unanswered question is whether deiminated peptide presentation also occurs during thymic development, and whether tolerizing peptides expressed by thymic antigen-presenting cells are also deiminated. In support of this possibility, we found that thymus lysates from B6 and B6.TC mice show abundant histone deimination (Figure 5E), a result that suggests antigen presentation in the mouse thymus is tightly linked to deimination. If so, citrulline-containing epitopes of autoantigens such as histones may induce powerful tolerance in mice. In that scenario, only B cells that bound to nH and presented non-deiminated epitopes would break tolerance and receive T cell help. Consequently, autoimmunity might initially be directed against non-deiminated peptides, provided that B cells, at an early stage of autoimmune pathogenesis, suspend or shut off the deimination of processed epitopes. Support for this alternative comes from the consistent anti-nH response that we observed in numerous autoimmune mice from different autoimmune strains (Figures 1 and 2). Thus, only B cells that no longer expressed PAD4 activity in their antigen processing compartment may escape tolerance. Our hypothesis of the key dependence of self-tolerance on the adequate function of autophagy for the presentation of dH peptides is in line with the remarkable preference of autoimmune mouse antibodies for nH. As corollary, a steady-state balance between nH and dH may be maintained under pre-autoimmune conditions, but an imbalance between the supply, processing, or recognition of nH, likely coincident with a disturbance of autophagic antigen processing in B cells, may result in an antigen-specific response to nH that may break immune tolerance and result in a sustained autoimmune response to nH.

To conclude, we briefly address the difference between mice and humans in their ability to express antibodies to dH. As we and others have shown, autoantibodies in various human autoimmune conditions preferentially bind dH (3, 24, 34), in striking contrast with the opposite bias in mice. Again, the key may be in the expression of PAD4 activity in B cells. Our tissue expression results indicate the increased presence of dH in the spleens of autoimmune mice (Figure 5B). By contrast, healthy human B cells appear incapable of expressing PAD4, as indicated by data in the Human Protein Atlas (35). There, evidence suggests that human B cells, even after B cell antigen engagement in the white pulp, fail to express detectable PAD4. Thus, expression of dH epitopes on human B cells may not be intrinsic to the B cells, and presentation of dH epitopes may not engender tolerance that it is as effective as it is in mice. Consequently, B cell presentation of deiminated peptides in humans may be more likely to break tolerance and lead to ACPA generation.

## Author Contributions

All authors were involved in drafting the article or revising it critically for important intellectual content, and approved the final version to be published. MR had full access to all the data in the study and took responsibility for the integrity of the data and the accuracy of data analysis. Study conception and design: ND, MS, LM, OR, and MR. Acquisition of data: ND, AH, YZ, IN, and MR. Analysis and interpretation of data: ND, AH, YZ, IN, MS, LM, OR, and MR.

## Conflict of Interest Statement

The authors declare that the research was conducted in the absence of any commercial or financial relationships that could be construed as a potential conflict of interest. The reviewers JD, EP, and GP and handling editor declared their shared affiliation, and the handling editor states that the process nevertheless met the standards of a fair and objective review.
